# Analysis of Nitrogen Dynamics and Transcriptomic Activity Revealed a Pivotal Role of Some Amino Acid Transporters in Nitrogen Remobilization in Poplar Senescing Leaves

**DOI:** 10.3390/plants12244140

**Published:** 2023-12-12

**Authors:** Min Zhou, Yuanlan Zhang, Jiading Yang

**Affiliations:** Co-Innovation Center for Sustainable Forestry in Southern China, College of Life Sciences, Nanjing Forestry University, Nanjing 210037, China; zm19981001@163.com (M.Z.); ylz@njfu.edu.cn (Y.Z.)

**Keywords:** nitrogen remobilization, leaf, autumnal senescence, gene expression, amino acid transporter

## Abstract

Leaf senescence is an important developmental process for deciduous trees during which part of leaf nitrogen is remobilized to branches, thus being beneficial for nitrogen conservation. However, the associated regulatory mechanism remains largely unknown in deciduous trees. In this study, nitrogen dynamics and transcriptomic activity in senescing leaves were measured during autumnal senescence in hybrid poplar. Both concentrations of leaf total nitrogen (N) and amine compounds were found to decline from the pre-senescence (PRE) to the middle-senescence (MS) stage. Although the total N concentration decreased further from MS to the late-senescence (LS) and leveled off to abscission (ABS) stage, amine compound concentration increased continuously from MS to ABS, suggesting that translocation of amine compounds underperformed production of amine compounds in leaves during this period. L-glutamate, L-glutamine and α-aminoadipic acid were the top three amine compounds accumulated in senescent leaves. RNA-Seq profiling identified thousands of differentially expressed genes (DEGs) with functional association with a metabolic transition towards disassimilation. Many genes encoding amino acid metabolism enzymes and amino acid transporters (AATs) were up-regulated. Comparison of expression trend with leaf N dynamics and phylogenetic analysis identified several *PtAATs* which exhibited down-regulation from MS to LS stage and putatively limited leaf N remobilization. This study can serve as a primary basis to further elucidate the molecular mechanisms of nitrogen remobilization in poplar senescing leaves.

## 1. Introduction

Senescence is the last developmental stage of leaves. Progressing of senescence is the integrative response of leaves to developmental age and various environmental factors [1]. Numerous studies have clearly shown that leaf senescence is regulated at multiple layers including chromatin remodeling and transcriptional, post-transcriptional, translational and post-translational regulations, thus making leaf senescence a highly coordinated process [2].

Different from plentiful studies using mutants in annual model plants, physiological and transcriptomic analysis were the main strategy for studying leaf senescence in perennial plants. In senescent leaves of *Populus tremula*, genes encoding metallothionein, early light-inducible proteins and cysteine proteases were most abundant [3], and transcripts related to photosynthetic light reactions and the Rubisco/Calvin cycle showed decreased expression, while transcripts related to mitochondrial respiration, oxidation of fatty acids and nitrogen mobilization (e.g., proteases and enzymes for amino acid metabolism) showed increased expression [4]. More recently, a high-resolution temporal profiling of transcripts during the autumnal leaf senescence process of *Populus tomentosa* was performed and 3459 autumn senescence-associated genes (SAGs) were identified and supposed to play important roles in metabolic pathways (such as carbohydrate, lipid and amino acid metabolism) and hormone responses [5]. However, only a few SAGs were functionally characterized in woody plants including poplar.

Accompanying the catabolism of macromolecules including chlorophylls, the most distinctive characteristic of leaf senescence is the orderly nutrient remobilization from leaves to other developing or storage organs, which is critical for plants’ productivity and fitness [6]. Among essential nutrients, nitrogen (N) is required by plants in the largest amount and limited N availability is frequently an environmental factor restricting plant growth and distribution [7]. Utilization of N by plants includes several steps, i.e., uptake, transport, assimilation and/or remobilization/reassimilation [8]. The efficiency of each step may contribute to a high nitrogen use efficiency (NUE) of plants. N remobilization out of leaves usually occurs when plants enter developmental senescence or precocious senescence induced by environmental stresses [9]. It has been shown that N remobilization during leaf senescence is fairly general for various plant species but with different efficiencies [10]. In annual crops, proteins in senescing leaves when plants enter the reproductive stage are extensively degraded and act as a nitrogen source for grain production. Delayed leaf senescence resulted in a less efficient N remobilization out of leaves and thus a declined grain protein content in wheat [11]. For perennial plants, especially such as deciduous trees during autumnal senescence, a portion of leaf nitrogen is remobilized to stems prior to leaf abscission [12,13]. From a physiological perspective, the N remobilized from senescent leaves to branches conserves nitrogen nutrients in trees and increases the cellular solute concentration thus improving the cold resistance of trees over winter, and N stored in branches could be utilized to support the new shoot growth in the next spring [14]. It has been shown that leaf nitrogen remobilization efficiency (NRE) of deciduous trees is not only associated with species types, but also influenced by environmental factors, such as geographic position/landform, soil nitrogen levels and water status [12,13,15]. In annual plants, improving leaf N remobilization through various transgenic approaches may enhance productivity and nitrogen use efficiency [16,17]. However, how N remobilization during yearly leaf senescence contributes to N budget and performance in deciduous trees is not well understood, mainly due to tree-specific characteristics such as large size, relatively slow growth and a long generation period [5].

Leaf N remobilization means that N-containing macromolecules (especially proteins) in leaves are degraded to soluble N-containing compounds (usually amino acids) which are then translocated either to young organs for reassimilation or to certain parts for storage. There are three sequential physiological steps, i.e., degradation of leaf proteins, interconversion of amino acids and phloem loading of certain amino acids, which together execute N transport out of senescing leaves [18]. Autophagy and proteolysis are thought to play a crucial role in protein degradation [19,20]. Amino acids resulting from protein degradation are converted to several specific ones (such as glutamine, glutamate, asparagine and serine, etc.), as they are usually the most abundant amino acids in the phloem sap of senescing leaves for N remobilization [18]. The interconversion of amino acids occurs in mitochondria and cytosol of mesophyll cells and companion cells and may be catalyzed by glutamate dehydrogenase (GDH), glutamate 2-oxoglutarate amino transferase/glutamate synthase (GOGAT), glutamine synthetase isoenzyme 1 (GS1) and asparagine synthetase (AS) [21]. Although either an apoplastic or a symplastic pathway may be functional for loading amino acids from the bundle sheath or vascular parenchyma cells into phloem, Arabidopsis and most crop plants are considered to be apoplastic phloem loaders [22]. Apoplastic loading involves two successive steps, i.e., amino acids first being released from source cells into extracellular space by passive transport and then imported into phloem pipelines via active transport [23]. The latter step is potentially performed by plasma membrane-localized amino acid transporters (AATs) [24] and is supposed to be rate-limiting for N transport from source leaves to sinks [25]. Identification of key AATs responsible for amino acid loading into phloem may be of great promise to improve the efficiency of N remobilization from senescing leaves.

Poplars are distributed worldwide and have high adaptability for various eco-environments [26]. Due to fast-growing and easy propagation by cuttings, hybrid poplar trees have long been considered as an important forestation species in China for ecological recovery [27]. Although some poplar species can grow in drought and nutrient-deficient soils, those fast-growing poplar trees normally require sufficient input of water and nitrogen fertilizer. However, the input of N fertilizer with increasing poplar forest area renders both ecological and economic disadvantages. Following lessons learned from studies about crop NUE and sustainable agriculture [28], breeding poplar varieties with improved nitrogen uptake and remobilization efficiency will be of great help to decrease fertilizer input while to maintain a sustainable development of poplar plantation forest.

In this study, the dynamics of leaf total N concentration and amine compound composition were measured during the autumnal leaf senescence of a hybrid poplar. The transcriptome analysis of leaves at various senescence stages was carried out to identify genes and processes associated with leaf N remobilization. The aim of this study was to uncover putative physiological process(s) restricting N remobilization from poplar senescing leaves and identify genes encoding amino acid transporters (AATs) which may be putatively utilized for improving N remobilization through ectopic overexpression.

## 2. Results

### 2.1. Concentrations of Chlorophyll and Total Nitrogen in Senescent Leaves

**Figure 1:**
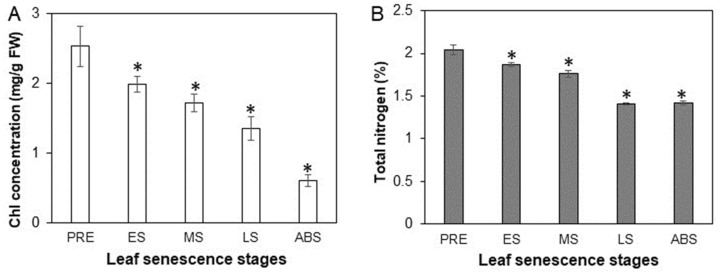
Changes of concentrations of Chl (**A**) and total nitrogen (**B**) in leaves at different senescence stages. Values are means ± SD (n = 3). The asterisks in each figure indicate the significant difference when the parameter at a certain stage compared to the immediate prior stage at *p* < 0.05, using Student’s *t*-test in Excel.

The leaf chlorophyll (Chl) concentration kept continuously declining from PRE to ABS, being 2.52 mg g^−1^ FW at the PRE stage, 1.98 at ES, 1.72 at MS, 1.34 at LS and 0.56 mg/g FW at the abscission stage. Compared to PRE, leaf Chl concentration at ABS decreased by about 76.6%. The total N concentration declined from 2.03% at PRE to 1.87% at ES, 1.76% at MS, to 1.41% at LS, and leveled off to the abscission stage (1.42%) (Figure 1), indicating that leaf total N did not decrease from LS to ABS stage. The percentage of N remobilized at ES, MS, LS and ABS compared to their immediate prior stage was estimated as 8.6%, 5.7%, 20.1% and −0.7% respectively, indicating that nitrogen remobilization from leaves was performed notably from MS to LS stage. Based on the leaf total N concentrations at PRE and ABS stages, the nitrogen remobilization efficiency (NRE) was calculated as 31%, which was much lower than the percentage of Chl concentration decreasing (76.6%) from PRE to ABS.

### 2.2. Fluctuation of Concentrations of Amine Compounds

**Figure 2:**
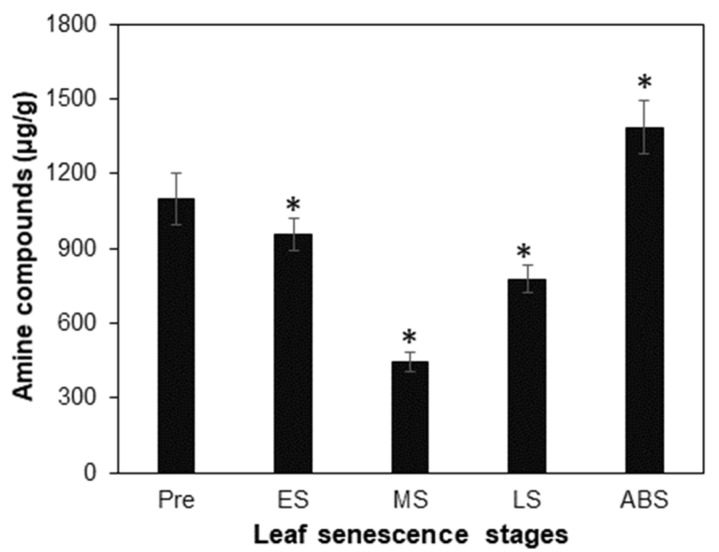
Concentrations of detected 57 amine compounds (including amine derivatives and amino acid compounds) at various leaf senescence stages. The * indicates a significant difference between the concentration at a certain stage and that at the immediate prior stage at *p* < 0.05, using Student’s *t*-test in Excel.

There were 57 amine compounds (including amine derivatives and amino acid compounds) that were detected in leaves at at least one stage (sheet 1, Supplemental Table S1). The concentration of amine compounds at five stages exhibited a V shape trend (Figure 2): i.e., being 1098 μg/g at the PRE stage, decreased by 13% at ES (as 955 μg/g) and decreased by 59% thus being lowest at MS (as 448 μg/g), both compared to PRE, then increased by 73% at LS (as 776 μg/g) and increased by 210% being highest at ABS (1387 μg/g), both compared to MS. Compared to the trend of leaf total N concentration (Figure 1B), it was found that concentrations of both amine compounds and total N in leaves showed a similar trend of decreasing from PRE to MS stage, while the total N concentration decreased from MS to LS and leveled off to ABS stage, the concentration of amine compounds increased continuously from MS to ABS. This result indicated that from MS to ABS, the rate of producing amine compounds in leaves exceeded that of amino acid translocating out of leaves, rendering an accumulation of free amine compounds in leaves.

Among 57 amine compounds, there were 8, 13, 12 and 17 members at ES, MS, LS and ABS stages, respectively, being differentially up- or down-regulated, compared to the PRE stage (Supplemental Table S1, sheet 2). When combined together, a total of 23 amine compounds were differentially up- or down-regulated at at least one stage after PRE, while the other 34 amine compounds were non-differentially regulated during leaf senescence (Supplemental Table S1). Considering that the concentration of 23 differential amine compounds at five stages exhibited a similar trend to and occupied the majority (88.2%, 87.0%, 79.5%, 87.5% and 90.5%, respectively) of that of all 57 amine compounds (Supplemental Figure S1); it was suggested that 23 differential amine compounds counted much for the fluctuation of amine compound concentrations during leaf senescence.

### 2.3. Main Differential Amine Compounds at Various Senescence Stages

The major members which constituted larger than 85% of the total concentration of 23 differential amine compounds at different senescence stages were similar but with distinctions (Table 1). For example, the major differential amine compounds at the PRE stage were L-glutamate, γ-aminobutyric acid, L-glutamine, L-aspartate and L-asparagine anhydrous, while at MS stage were L-glutamate, L-glutamine, γ-aminobutyric acid, L-tryptophan, L-aspartate, glutathione oxidized, α-aminoadipic acid, argininosuccinic acid and creatine phosphate. Notably, L-glutamate and L-glutamine were among the top three differential amine compounds at either of the senescence stages, while α-aminoadipic acid was among the top three differential amine compounds only at the LS and ABS stages. The combined concentration of L-glutamate and L-glutamine represented 64.3%, 59.7%, 57.4%, 68.0% and 65.5% of the concentration of all differential amine compounds at PRE, ES, MS, LS, and ABS, respectively. Especially, the concentrations of L-glutamate at LS and ABS stages were 2.2 and 3.0 times,, respectively, of that at the MS stage, while L-glutamine concentrations at LS and ABS stages were 2.4 and 6.7 times, respectively, of that at the MS stage. These results indicated that L-glutamate, L-glutamine and α-aminoadipic acid may be mainly responsible for the increased concentration of amine compounds in poplar senescing leaves before the ABS stage.

### 2.4. Differentially Expressed Genes during Leaf Senescence

The number of clean reads generated from each of the 12 cDNA libraries (including biological replicates) ranged from 43.08 to 56.70 million. Approximately 76.68% to 85.17% of clean reads per cDNA library could be mapped to the reference genome of *P. trichocarpa* and a total of 41,298 transcripts were obtained. Based on multiple criteria (see Materials and Methods), 8955 transcripts were selected as differentially expressed genes (DEGs, Table S2 available as Supplementary Data online). Among them, 4605 DEGs that showed up-regulation (expression fold change ≥ 2.0 compared to expression at PRE) at at least one stage of ES, IS or LS were regarded as senescence-induced genes (SIGs), while other 4350 genes with only decreased expression (fold change ≤ 0.5) at any stages after PRE stage were regarded as senescence-downregulated genes (SDGs).

**Figure 3:**
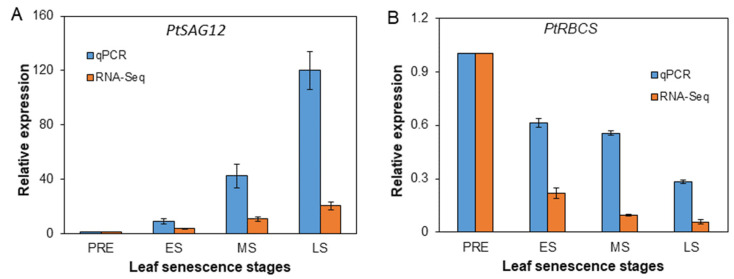
Relative expression of two marker genes of leaf senescence measured by qPCR and RNA-Seq respectively. (**A**) *PtSAG12* (Potri.004g055900); (**B**) *PtRBCS* (Potri.004g100000).

The expressions of a cysteine protease encoding gene (PtSAG12) and a ribulose biphosphate carboxylase small subunit encoding gene (PtRBCS), which are widely used as marker genes of leaf senescence [29], were measured and compared to expression levels calculated using FPKM values in RNA-Seq results with PRE as control The trends of expression from PRE to LS stage measured by qPCR and by RNA-Seq were very similar, i.e., being continuously increasing with the maximum expression at LS stage for *PtSAG12* while being continuously decreasing with the lowest expression at LS stage for *PtRBCS* (Figure 3A,B), thus indicating the sequential advancing of leaf senescence and reliability of the RNA-Seq results.

**Figure 4:**
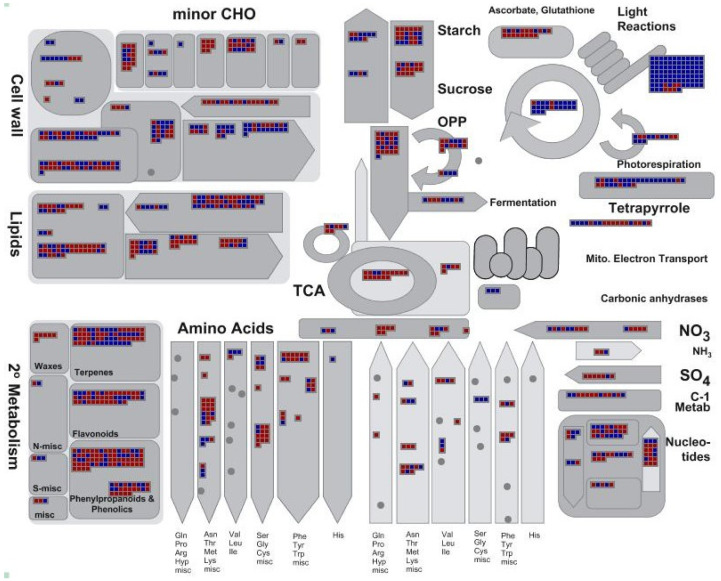
Distribution of differentially expressed genes (DEGs) in various metabolic pathways during leaf senescence, viewed by MapMan software. DEGs were represented by red and blue squares in the figure, with red standing for up-regulation and blue standing for down-regulation. Those genes participating in selected metabolic processes were listed in Supplemental Table S3.

To understand the functional association of 8955 DEGs with leaf senescence, MapMan analysis (Pathways: Metabolism_overview) [30] was performed. As shown in Figure 4 and Supplemental Table S3, most of the DEGs involved in photosynthesis (i.e., 85 out of 91 DEGs for light reactions and 20 out of 23 DEGs for the Calvin cycle) showed down-regulation. Although most of DEGs involved in sugar synthesis (i.e., 7 out of 10 for starch synthesis and 3 out of 4 for sucrose synthesis) were down-regulated, most of DEGs participating in sugar degradation (i.e., 15 out of 24 for starch degradation, 13 out of 16 for sucrose degradation, 17 out of 26 for glycolysis, 18 out of 21 for tricarboxylic acid (TCA) cycle and organic acid transformation) showed up-regulation. Especially, there were five gene encoding isocitrate dehydrogenase (i.e., Potri.004g074900, Potri.005g099600, Potri.010g176000, Potri.017g144500 and Potri.006g126700) in the TCA cycle catalyzing production of 2-oxoglutarate being up-regulated (Supplemental Table S3). As for lipid degradation, 30 out of 45 related DEGs showed upregulation. There were 12 out of 18 DEGs participating in nucleic acid degradation being upregulated. Especially, protein degradation pathways (both ubiquitin- and autophagy-dependent) contained 272 DEGs, out of which 156 showed up-regulation. Notably, all 18 DEGs for proteasome and all 3 DEGs for autophagy showed up-regulation (sheet “protein degradation” of Supplemental Table S3). Similarly, DEGs involved in secondary metabolism (including waxes, terpenes, flavonoids, phenylpropanoids and simple phenols) showed more up-regulated genes than down-regulated (132 vs. 70). These results collectively suggested a general transition of primary metabolism from assimilation towards disassimilation which was paralleled with active secondary metabolism in poplar leaves during senescence process.

### 2.5. Expression Patterns of SIGs Involved in Amino Acid Interconversion

**Figure 5:**
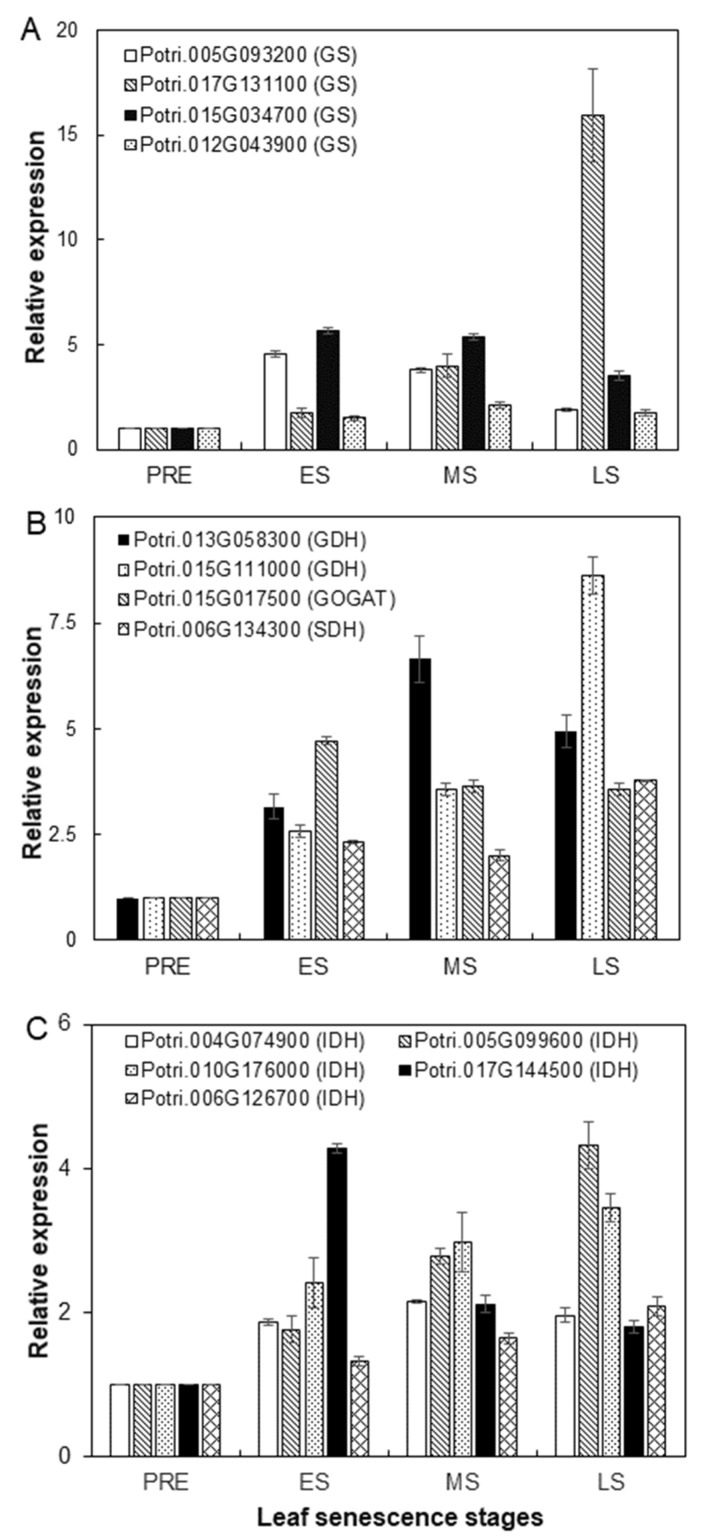
Expression of senescence-induced genes which were annotated to encode enzymes responsible for biosynthesis of L-glutamine, L-glutamate and α-aminoadipic acid. (**A**) four SIGs encoding glutamine synthetase (GS). (**B**) SIGs encoding two glutamate dehydrogenase (GDH), one glutamate synthase (GOGAT), and one saccharopine dehydrogenase (SDH), respectively. (**C**) five SIGs encoding isocitrate dehydrogenase (IDH). The FPKM values were extracted from RNA-Seq results (Supplemental Table S2) and relative expression at ES, MS and LS stages was calculated relative to expression at PRE (n = 3).

Considering that only several specific amine compounds (such as L-glutamate, L-glutamine and α-aminoadipic acid, etc.) were mainly accumulated in poplar senescing leaves before ABS stage (Table 1), SIGs encoding enzymes catalyzing biosynthesis of these amine compounds, i.e., glutamine synthetase (GS), glutamate dehydrogenase (GDH), and glutamate synthase (GOGAT) [31], were searched based on gene annotations (Supplemental Table S4). As shown in Figure 5A, four GS genes (Potri.005G093200, Potri.017G131100, Potri.015G034700 and Potri.012G043900) were identified which exhibited respective peak expression at various stages. The expression of Potri.017G131100 showed a continuous increase during senescence with the highest expression at LS. As shown in Figure 5B, two GDH genes (Potri.013G058300 and Potri.015G111000) exhibited their peak expression at MS and LS, respectively, while one GOGAT gene (Potri.015G017500) was up-regulated during senescence with peak expression at MS. Additionally, one gene encoding a saccharopine dehydrogenase (SDH), which may participate in α-Aminoadipic acid biosynthesis [32], was also identified with expression peak at LS stage. 2-oxoglutarate, whose production is catalyzed by isocitrate dehydrogenase (IDH) in the TCA cycle, is the substrate for glutamate production through reductive amination with either ammonium or glutamine as the nitrogen source [33]. Five IDH-encoding genes were identified among SIGs (Figure 5C). Three of them (i.e., Potri.005G099600, Potri.010G176000 and Potri.006G126700) exhibited continuously increased expression from PRE to LS stage, putative indicating the association of sugar metabolism with amine compound production.

### 2.6. Identification of Target SIGs Encoding Amino acid Transporters (AATs)

**Figure 6:**
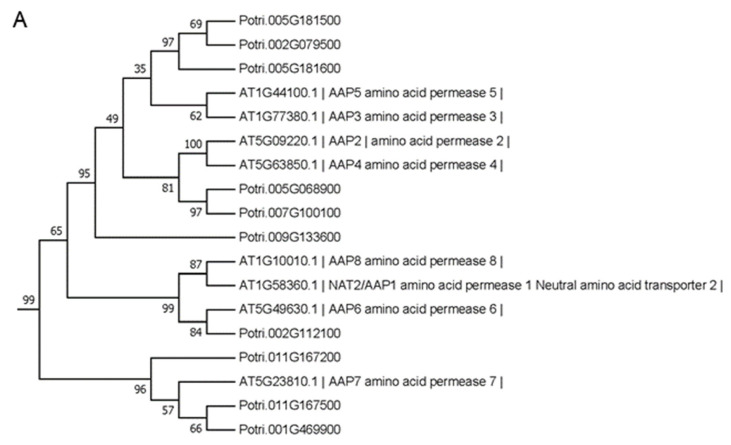

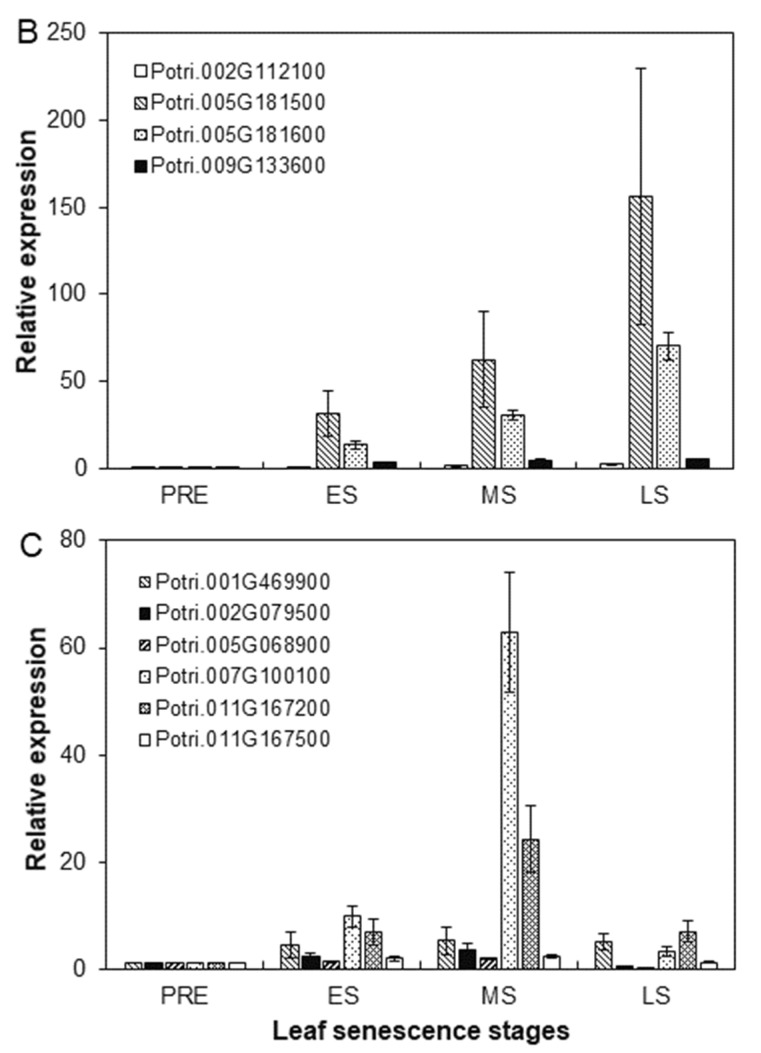
Identification of target SIGs encoding amino acid transporters (AATs). (**A**) The target subclade of the phylogenetic tree containing all 8 Arabidopsis amino acid permeases (AAPs) and 10 PtAAT proteins (the whole phylogenetic tree was presented in Supplemental Figure S2). (**B**) Expression patterns of 4 *PtAATs* with increased expression from MS to LS stage. (**C**) Expression patterns of 6 *PtAATs* with decreased expression from MS to LS stage. The FPKM values of each gene were extracted from RNA-Seq results and relative expression at ES, MS and LS stages was calculated relative to expressions at PRE stage.

Among 4605 SIGs, a total of 34 members were putative *PtAATs* in poplar, according to a previous report (Supplemental Table S4) [34]. The protein sequences of these 34 PtAATs, together with 66 Arabidopsis AATs were submitted to phylogenetic analysis. It was found that 34 PtAATs were distributed in multiple subclades in the phylogenetic tree (Supplemental Figure S2), putatively indicating their diverse functions during leaf senescence. Considering that amino acid permeases (AAPs), capable of transporting acid and neutral amino acids, are supposed to be the primary amino acid phloem loaders in plants [35], the subclade containing all 8 AtAAPs and 10 PtAATs was selected to check their expression pattern (Figure 6A). While 4 *PtAATs* exhibited increasing of expression from MS to LS stage (Figure 6B), expression of other 6 *PtAATs* (i.e., Potri.001G469900, Potri.002G079500, Potri.005G068900, Potri.007G100100, Potri.011G167200 and Potri.011G167500) decreased from MS to LS stage (Figure 6C), which was just opposite to increasing of the concentration of amine compounds from MS to LS stage (Figure 2). Based on the reasoning that down-regulation of key *PtAATs* may result in a limitation of amino acid translocation out of leaves, thus causing an accumulation of amino acids, these 6 PtAATs were indicated as targets of priority to be characterized for their exact function of transporting amino acids into phloem.

## 3. Discussion

Senescence as the last stage of leaf development consists of a series of highly controlled physiological events of disassembly and degeneration [6]. Leaf phenotypic yellowing during senescence due to Chl degradation indicates the transition of leaf function from an assimilative photosynthetic organ to a disassimilative source organ, which then provides nutrients to other growing organs or storage organs [18]. Declining of Chl concentration is the most frequently used physiological trait characterizing leaf senescence progression [36], although certain genetic mutations disrupting Chl degradation may retard Chl but without delaying the drop of leaf photosynthetic efficiency during senescence [37]. In this study, Chl concentration exhibited a continuous decline, and about 76.6% of Chl at PRE was lost at the ABS stage (Figure 1A). However, although leaf total N concentration decreased from PRE to LS, it leveled off from LS to ABS (Figure 1B), thus exhibiting a different trend compared to Chl concentration. The nitrogen remobilization efficiency (NRE), i.e., the percentage of leaf total N at PRE being translocated at ABS, was 31%, which was much lower than the estimated global leaf NRE (44–56%) of woody plants [38]. Although leaf NRE of a certain plant may be influenced by environmental conditions (such as geographical location, soil fertility, precipitation, and temperature) [39], at least in this study, constant concentration of leaf total N from LS to ABS indicated a potential possibility of improving NRE of hybrid poplar trees by promoting N translocation out of leaves after LS stage. Considering that the primary burst of leaves and or flowers in spring of deciduous trees depends mainly on nitrogen stored in branches [12]; theoretically, the more leaf N remobilized in the previous autumn, the more N stored in branches available for new shoot growth, thus somehow decreasing demand of N fertilizer input for a tree in next growth season. Especially for a large area of plantation forest, improving nitrogen remobilization efficiency may have great potential to save N fertilizer input, thus decreasing management costs and being beneficial for sustainable forestry development.

Amino acids are the main form of organic N remobilized from senescent source leaves in most plant species [22,23,40]. In this study, although concentrations of amine compounds and total N in senescing leaves both decreased from PRE to MS, the concentration of amine compounds increased from MS to ABS which was accompanied by leaf total N concentration decreasing from MS to LS and leveling off to ABS (Figure 1B and Figure 2). The different patterns of concentrations of amine compounds and total N during poplar leaf senescence putatively indicated that the rate of translocation of amine compounds out of leaves was lower than the rate of production, thus causing an increased concentration of amine compounds in leaves before abscission. Interestingly, along with the increasing leaf age, the concentration of amino acids was found to increase gradually in Arabidopsis leaves but decrease constantly in oat leaves [41]. For 11-week-old tobacco plants, amino acid concentration in leaves from top to bottom position (i.e., with increasing age) showed a progressive decline [42]. However, in tobacco plants, the concentration of a total of 22 proteinogenic and nonproteinogenic amino acids exhibited a gradual increase in middle leaves after 45 DAT (days after topping) and in upper leaves after 55 DAT, respectively [43]. These results collectively indicated that differential dynamics of amine compound concentration in senescing leaves may be variable with plant species, leaf senescence type (e.g., position-related or development-associated) and experimental condition. But it is of interest to monitor the concentration of amine compounds in senescing leaves of more deciduous trees to know if the accumulation of amine compounds is a general physiological phenomenon across woody plants.

Leaf senescence is strictly controlled by multiple layers of regulation accompanied by extensive changes in gene expression [1,6]. In this study, transcriptomic profiling of poplar leaves at sequential stages identified thousands of genes with differential expression (DEGs) compared to the pre-senescence stage (Supplemental Table S2). Metabolism_overview by MapMan software clearly showed that more DEGs participating in the degradation of sugars, lipids, proteins and nucleic acids were up-regulated, while more DEGs participating in photosynthesis (including light reaction and Calvin cycle) were down-regulated (Figure 4, Supplemental Table S3). Consistent with previous studies [4,44], such a change of transcriptomic activity underlay the functional transition of poplar leaves from assimilation towards degeneration, thus becoming a source organ providing nutrients for remobilization. The similar physiological transition of leaf function during senescence in poplar and Arabidopsis indicated that leaf autumnal senescence of perennial woody trees shares substantial common mechanisms with leaf senescence of annual herbaceous plants [44].

Profiling phloem sap collected from senescing leaves of tobacco, Arabidopsis and other plants showed that not every amino acid resulting from protein degradation is remobilized equally, as the most abundant amino acids in phloem sap are mainly glutamine, asparagine, glutamate, aspartate and serine, with variations depending on plant species and growth conditions [18,21], thus highlighting the involvement of amino acid interconversion during N remobilization. In this study, it was found that L-glutamate, L-glutamine and α-aminoadipic acid were the top three compounds whose concentrations exhibited a substantial increase from the MS to ABS stage (Table 1), which may underlie the increased concentration of all amine compounds in leaves during the same period (Figure 2, Supplemental Figure S1). The increased concentration of these three compounds may indicate that the rate of their synthesis exceeded the rate of their translocation out of leaves. In plants, the couple of glutamine synthetase (GS) and glutamate synthase (GOGAT), known as the GS/GOGAT cycle, which assimilates ammonium into glutamine by GS and then converts glutamine and 2-oxoglutarate derived from the TCA cycle into two molecules of glutamate by GOGAT, is the predominant route for the synthesis of glutamine and glutamate [21,45]. In addition, glutamate dehydrogenase (GDH) may aminate 2-oxoglutarate to form glutamate under stress conditions or during leaf senescence to prevent cellular ammonium toxicity [43,46]. In agreement with the accumulation of glutamate and glutamine in poplar leaves after the MS stage (Table 1), four *GSs*, two *GDHs* and one *GOGAT* were found to be up-regulated during poplar leaf senescence (Figure 5A,B). Meanwhile, five genes encoding isocitrate dehydrogenase (IDH) in the TCA cycle were up-regulated (Figure 5C, Supplemental Table S3), which might provide enough 2-oxoglutarate as carbon skeletons for the synthesis of glutamate and glutamine [47]. Although asparagine was found to be an essential compound for N storage and transport in numerous plants [48], it was only present as a major compound at the PRE, ES and ABS stages with concentration proportions less than 5%, indicating that asparagine may play a less important role than glutamate or glutamine in N remobilization in poplar leaves (Table 1). At present, it remains unknown what physiological or molecular mechanism underlies such a specific composition of amine compounds in poplar senescent leaves.

In Arabidopsis and most crop plants, amine compounds produced in cells of senescing leaves are believed to be released first into extracellular space by passive transport and then imported into sieve element/companion cell complexes by plasma membrane-localized amino acid transporters (AATs) via ATP-consuming active transport [23,24]. Pumping amino acid compounds by AATs into phloem is the rate-limiting step for N remobilization out of senescent leaves [25]. Although there are hundreds of putative AATs identified by bioinformatic analysis in different plant species [23], only a small portion was characterized to be involved in various processes of amino acid transport respectively, including root uptake and xylem loading, mesophyll cell uptake, xylem-phloem exchange, phloem loading and unloading, sink organ loading, etc. [49]. In this study, 34 *PtAATs* were identified in SIGs (Supplemental Table S4) and 34 PtAATs were distributed in multiple subclades of the phylogenetic tree with Arabidopsis AATs of various subfamilies (Supplemental Figure S2). In Arabidopsis, AMINO ACID PERMEASE 8 (AAP8) was shown to be expressed in the phloem of source leaves and is capable of transporting both acidic and neutral amino acids, including glutamate and glutamine [50]. Loss of function of AAP8 in the aap8 mutant resulted in decreased phloem loading of ^14^C-labeled glutamate or glutamine fed to the source and thus decreased partitioning to sink leaves and siliques [50]. In pea (*Pisum sativum*, cultivar ‘Bohatyr’), overexpression of the endogenous AAP1 transporter in the leaf phloem (driven by Arabidopsis *AAP1* promoter) resulted in an increased phloem loading and embryo loading of amino acids, thus improving seed yield [51]. Based on the increased concentrations of amine compounds from MS to LS stage (Figure 2), it was indicated that the rate of remobilizing was lower than that of producing amine compounds in poplar senescing leaves, which may be attributed putatively to the downregulation of some key *PtAATs*. Among 10 PtAATs together with all 8 AtAAPs grouped in the same phylogenetic subclade (Figure 6A), the expression of 6 *PtAATs* decreased from the MS to the LS stage (Figure 6C). Thus, it was reasonable to ratiocinate that downregulation of these six *PtAAPs* might limit phloem loading of amine compounds in poplar senescing leaves before the ABS stage and their exact function may deserve further characterization in the future. Additionally, although current knowledge suggests that AAPs are the primary amino acid phloem loaders in plants, certain members of other transporter families might also be functional in the phloem loading of amino acids [35].

In summary, the chlorophyll concentration in poplar leaves exhibited a continuous decrease during autumnal senescence, while the concentration of leaf total N decreased from pre-senescence to late-senescence and leveled off to the abscission stage. The concentration of amine compounds declined from pre-senescence to middle-senescence and increased from middle-senescence to abscission stage. The most accumulated amine compounds were L-glutamate, L-glutamine and α-aminoadipic acid. Thousands of genes with functional association with a metabolic transition towards disassimilation were differentially expressed during leaf senescence. Multiple genes encoding N remobilization-related genes (i.e., those encoding amino acid metabolism enzymes and amino acid transporters (AATs)) were upregulated. Phylogenetic and expression pattern analysis identified six PtAAT genes encoding amino acid permeases are promising targets limiting amino acid remobilization out of poplar senescing leaves. This study may serve as a starting point for further elucidating molecular mechanisms of leaf nitrogen remobilization during autumnal senescence in poplar and other deciduous trees.

## 4. Materials and Methods

### 4.1. Plant Materials and Sample Collection

From late September to middle November (about leaf abscission) in 2018, three healthy one-year-old branches were cut around 10:00 AM every week at middle position of outside canopy of a hybrid poplar tree (*Populus deltoides* × *P. euramericana* cv. ‘Nanlin895’) which grew naturally on the campus of Nanjing Forestry University, Nanjing, China (34°4′ N latitude and 118°48′ E longitude). The two or three newly expanded top leaves from each branch were collected as one biological replicate and frozen in liquid N, then stored at −80 °C until further processing, thus there were three biological replicates at each sampling time point.

### 4.2. Physiological Analysis

Leaf samples were ground into fine powder in liquid N using a freezer mill and submitted for analysis of chlorophyll (Chl) and amine compounds. The Chl concentration was determined by measuring absorption at 663 and 646 nm [52]. Based on the criterion that Chl concentration in leaves at a later time point was lower significantly than that at the immediately prior time point, five time points, i.e., 29 September, 13 October, 27 October, 3 November and 16 November were selected as pre-senescence, early-, middle-, late-stage of senescence and abscission stages for poplar leaves in this study (abbreviated as PRE, ES, MS, LS and ABS, respectively).

For measuring the concentration of total nitrogen, the leaf samples at five stages were dried at 65 °C until their dry weight was stable (at least 48 h) and ground into a fine powder using mortar/pestle. Powder samples were digested following Kjeldahl’s method (using H_2_SO_4_-H_2_O_2_) and the concentration of N in the digested solution after dilution was determined using PerkinElmer 2400 Series II CHNS/O Analyzer (PerkinElmer Inc., Waltham, MA, USA) and the total N in leaf samples (%) was calculated. The percentage of remobilized N at either of the ES, MS, LS and ABS stages was estimated relative to its immediate prior stage (i.e., [(N_latter_ − N_prior_)/N_prior_]). The NRE was calculated according to the leaf N concentrations at PRE and ABS stages as [(N_PRE_ − N_ABS_)/N_PRE_] × 100%.

For measurement of N-containing metabolites, leaf samples stored at −80 °C were freeze-dried by vacuum freeze-dryer (Scientz-100F, Shanghai Yetuo Technology Co., Ltd., Shanghai, China) and were crushed using a mixer mill (MM 400, Retsch, Scientific Industries, Bohemia, NY, USA) with a zirconia bead for 1.5 min at 30 Hz. Each ground sample (0.05 g) was mixed with 500 µL of 70% methanol/water (containing 0.1 mg/L lidocaine as internal standard). The solution was vortexed for 5 min and centrifuged at 10,000× *g* for 10 min at 4 °C. An aliquot of 200 μL supernatant was filtrated (SCAA-104, 0.22-μm pore size; ANPEL, Shanghai, China) for further LC-MS analysis. According to the method reported previously [53], UPLC and ESI-Q TRAP–MS/MS were performed, and N-containing metabolites (including amine derivatives and amino acid compounds) were detected based on the AB Sciex QTRAP 6500 LC-MS/MS platform by Metware Biotech. Co., Ltd. (Wuhan, China). An amine compound with both a value of the variable important difference (VIP) ≥ 1 and fold change ≥ 2.0 (up) or ≤0.5 (down) if its concentration at either of the latter stages was compared to that at PRE stage (as control) was regarded as significantly up- or down-regulated [54].

### 4.3. Transcriptome Sequencing, Sequence Assembly and Functional Annotation

Leaf samples collected at four stages (i.e., PRE, ES, IS and LS) except ABS were submitted for RNA-Seq, because no high-quality RNA could be extracted from samples at ABS stage. Total RNA samples were extracted using the TRIzol reagent (Invitrogen) and then treated with RNase-free DNase I (Takara, Beijing, China) to remove genomic DNA. The quantity and quality of RNAs were tested using a Nanodrop 2000 spectrophotometer (NanoDrop Tchnologies, Wilmington, ED, USA) and Agilent Bioanalyser 2100 system (Agilent Technologies, Palo Alto, CA, USA), respectively. The mRNAs were first purified from RNA samples using poly-T oligo-attached magnetic beads. Strand-specific RNA-Seq library preparation and sequencing were both performed by Novogene Biotech (Beijing, China) using standard Illumina protocols on an Illumina HiSeq 2500 system with the paired-end mode. Adaptor sequences and low-quality sequences were removed from the raw reads (Q < 20). Clean reads were assembled using the Cufflinks v2.1.1 reference annotation-based transcript (RABT) assembly method [55] and mapped to the reference genome of *Populus trichocarpa* (https://phytozome.jgi.doe.gov/pz/portal.html accessed on 17 August 2019) to get gene annotations using HISAT (2.1.0) software [56].

### 4.4. Selection of Differentially Expressed Genes (DEGs), qPCR Validation and Functional Overview

The FPKM (reads per kb per million reads) was used to eliminate the influence of different gene lengths and sequencing discrepancies on the quantification of gene expression to ensure direct comparison of gene expression in different sample pairs. A gene was classified as ‘detected’ if the average FPKM of three biological replicates was ≥1.0 for at least one stage. The variation of gene expression during leaf senescence was calculated by taking expression at the PRE stage as the control and using the DESeq package (v1.10.1) [57]. Differentially expressed genes (DEGs) were determined by using a false discovery rate (FDR)-adjusted *p*-value (Padj) less than 0.05 and the absolute value of log2 (Fold change of expression) ≥ 1 at any stages after PRE. DEGs with ≥2 folds up-regulation on at least one stage of ES, LS and ABS were regarded as senescence-induced genes (SIGs), while DEGs with only decreased expression (fold change ≤ 0.5) at stages after the PRE stage were regarded as senescence-downregulated genes (SDGs).

Quantitative real-time PCR (qPCR) was performed to measure the transcript levels of two selected senescence marker genes in leaf samples used for RNA-Seq. The first-strand cDNA was synthesized with 1 μg of total RNA using the Evo M-MLV Reverse Transcription kit (Accurate Biology, Changsha, China), and quantitative real-time PCR (qPCR) was fulfilled using SYBR Green Pro Taq HS Premix (Accurate Biology) with a QuantStudio 3 Real-Time PCR System (Applied Biosystems, Foster City, CA, USA). The qPCR procedure involved 95 °C hold for 15 min followed by 40 cycles at 95 °C for 10 s and 60 °C for 35 s. Two selected senescence marker genes included a cysteine protease encoding gene (SAG12 homolog in poplar, *PtSAG12*, Potri.004g055900) and a ribulose biphosphate carboxylase small subunit encoding gene (RBCS in poplar, *PtRBCS*, Potri.004g100000) [29]. A *Ubiquitin* gene of *Poplus trichocarpa* (*PtUBQ*, Potri.011G134200) which showed stable expression during leaf senescence was used as the internal reference gene for normalization. The transcript levels of selected genes at the latter stages relative to that at PRE stage were calculated using the equation 2^−ΔΔCt^, where Ct is the threshold cycle for each gene. The primers for *PtUBQ* were 5′-CCACCCTTCACCTTGTCCT-3′ (forward) and 5′-AAATGGCTACTGAGCACACACA-3′ (reverse); for *PtSAG12* were 5′-GGGTGAAAGTGGGTATATGAGG-3′ (forward) and 5′-TCATGCAGTTGGATAAGAAGCA-3′ (reverse); for *PtRBCS* were 5′-GGGTACTATGATGGACGCTACTG-3′ (forward) and 5′-CTCCTCAAGCTCAAGCAACAC-3′ (reverse).

To know the metabolic functions of DEGs, MapMan software (version 3.6.0RC1) [30] (Pathway: Metabolism_overview; and Proteasome Detail And Autophagy) was utilized using the mapping file Ptrichocarpa_v3.0_210_peptide.txt (https://mapman.gabipd.org/mapmanstore (accessed on 11 December 2023)). The expression value was simply set to 1 for SIGs and −1 for SDGs to inspect the expression status (up- or down-regulated) of DEGs in various metabolic pathways.

### 4.5. Annotation Searching for N Remobilization-Related Genes

Annotations of SIGs were searched for genes encoding key enzymes of amino acid metabolism [i.e., glutamate dehydrogenase (GDH), glutamine synthetase (GS) and glutamate 2-oxoglutarate amino transferase/glutamate synthase (GOGAT), isocitrate dehydrogenase (IDH)] [21], as well as saccharopine dehydrogenase (SDH) which may participate in α-aminoadipic acid biosynthesis [33]. The FPKM values of selected genes were used to calculate their relative expression at ES, MS and LS stages with PRE as control.

### 4.6. Phylogenetic Analysis of Amino Acid Transporters

Based on the previous report which revealed 100 putative amino acid transporters (AATs) in the *Populus trichocarpa* genome [34], the SIGs encoding putative AATs (i.e., PtAATs) were identified in this study. The protein sequences of PtAATs were submitted to phylogenetic analysis with known Arabidopsis AtAATs [24,58], thus identifying putative target PtAATs which may play a critical role in N remobilization of senescing leaves. All peptide sequences were input to MAFFT (online version) (https://mafft.cbrc.jp/alignment/server/index.html (accessed on 24 November 2023)) to perform alignment with default parameter values. The alignment results were used to produce a maximum likelihood phylogenetic tree using MEGA7. The Bootstrap method, with 1000 replications, was used to provide confidence levels (reported as a percentage) of branch points on the phylogenetic tree. Those PtAATs present with AtAATs with known functions of translocating specific amino acids in the same subclade of the phylogenetic tree were regarded as putative targets.

## Figures and Tables

**Table 1:** The major differential amine compounds at various senescence stages.

Compounds with Concentration from High to Low	PRE	ES	MS	LS	ABS
1	L-glutamate	L-glutamate	L-glutamate	L-glutamate	L-glutamate
	533.57 (55.1%)	364.10 (43.8%)	149.78 (42.0%)	330.71 (48.5%)	455.12 (36.2%)
2	γ-aminobutyric acid	L-glutamine	L-glutamine	L-glutamine	L-glutamine
	119.55 (12.3%)	133.36 (16.0%)	55.10 (15.4%)	132.54 (19.5%)	367.62 (29.3%)
3	L-glutamine	γ-aminobutyric acid	γ-aminobutyric acid	α-aminoadipic acid	α-aminoadipic acid
	88.89 (9.2%)	62.26 (7.5%)	19.64 (5.5%)	30.11 (4.4%)	98.82 (7.9%)
4	L-aspartate	L-aspartate	L-tryptophan	glutathione oxidized	L-tryptophan
	63.83 (6.6%)	46.84 (5.6%)	18.70 (5.2%)	28.86 (4.2%)	94.69 (7.5%)
5	L-asparagine anhydrous	L-asparagine anhydrous	L-aspartate	L-aspartate	γ-aminobutyric acid
	40.55 (4.2%)	40.65 (4.9%)	16.74 (4.7 %)	27.29 (4.0%)	49.87 (4.0%)
6	others together	L-tryptophan	glutathione oxidized	L-tryptophan	L-asparagine anhydrous
	122.25 (12.6%)	35.44 (4.3%)	16.58 (4.6%)	25.33 (3.7%)	31.12 (2.5%)
7		creatine phosphate	α-aminoadipic acid	γ-aminobutyric acid	others together
		31.60 (3.8%)	13.68 (3.8%)	23.55 (3.5%)	158.31 (12.6%)
8		others together	argininosuccinic acid	others together	
		117.48 (14.1%)	12.21 (3.4%)	82.84 (12.2%)	
9			creatine phosphate		
			11.55 (3.2%)		
10			others together		
			42.87 (12.0%)		

Note: Below compounds, the numbers correspond to the average concentration (n = 3) and the numbers between parentheses correspond the concentration percentage of all 23 differential amine compounds at each senescence stage: PRE, Pre-senescence; ES, Early-senescence; MS, Middle-senescence; LS, Late-senescence; and ABS, Abscission stage.

## Data Availability

The Raw FASTQ files of RNA-Seq results have been deposited in the China National Genomics Data Center with BioProject accession number PRJCA020665 (https://ngdc.cncb.ac.cn (accessed on 11 December 2023)).

## Supplementary Materials

The following supporting information can be downloaded at: https://www.mdpi.com/article/10.3390/plants12244140/s1, Figure S1. Concentrations of 23 differential and 34 non-differential amine compounds at various senescence stages. Figure S2. The intact phylogenetic tree of 34 PtAATs and 66 Arabidopsis AATs. Table S1. Amine compounds detected in poplar senescing leaves at five stages. Table S2. List of differentially expressed genes. Table S3. List of differentially expressed genes participating in various MapMan pathways. Table S4. Senescence-induced genes encoding enzymes for amino acid interconversion and amino acid transporters; IDs of 66 AtAATs and 10 PtAATs in the selected subclade of the phylogenetic tree.
