# Supplementary material for: Analysis of Nitrogen Dynamics and Transcriptomic Activity Revealed a Pivotal Role of Some Amino Acid Transporters in Nitrogen Remobilization in Poplar Senescing Leaves
Source: Plants (Basel). 2023 Dec 12;12(24):4140. doi: 10.3390/plants12244140 (PMC10747403; doi:10.3390/plants12244140)
Supplement: Supplementary file 1 [file plants-12-04140-s001.zip › Supplemental Figure S1.pdf]

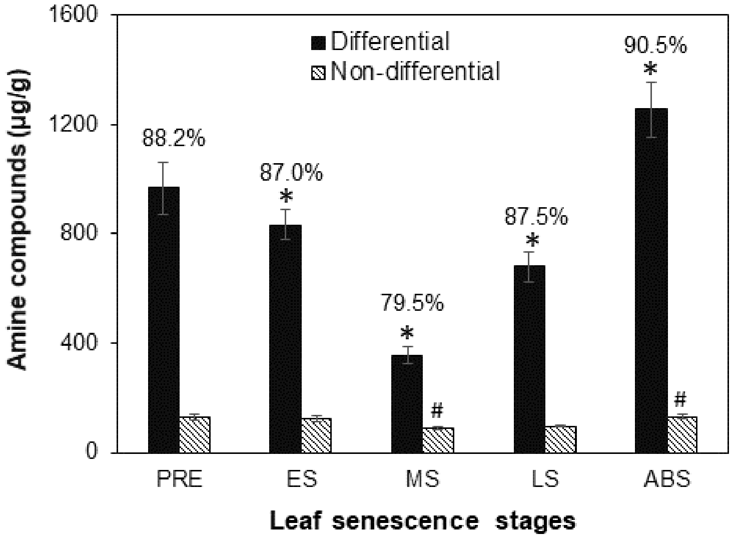

Supplemental Figure S1. Concentrations of 23 differential and 34 non-differential amine compounds at various senescence stages. A significant difference (at $p < 0.05$) between the concentration at a certain stage and that at the immediate prior stage is indicated by * (for differential compounds) and # (for non-differential compounds), using Student's *t*-test in Excel.
