# Supplementary material for: Analysis of Nitrogen Dynamics and Transcriptomic Activity Revealed a Pivotal Role of Some Amino Acid Transporters in Nitrogen Remobilization in Poplar Senescing Leaves
Source: Plants (Basel). 2023 Dec 12;12(24):4140. doi: 10.3390/plants12244140 (PMC10747403; doi:10.3390/plants12244140)
Supplement: Supplementary file 1 [file plants-12-04140-s001.zip › Supplemental Figure S2.pdf]

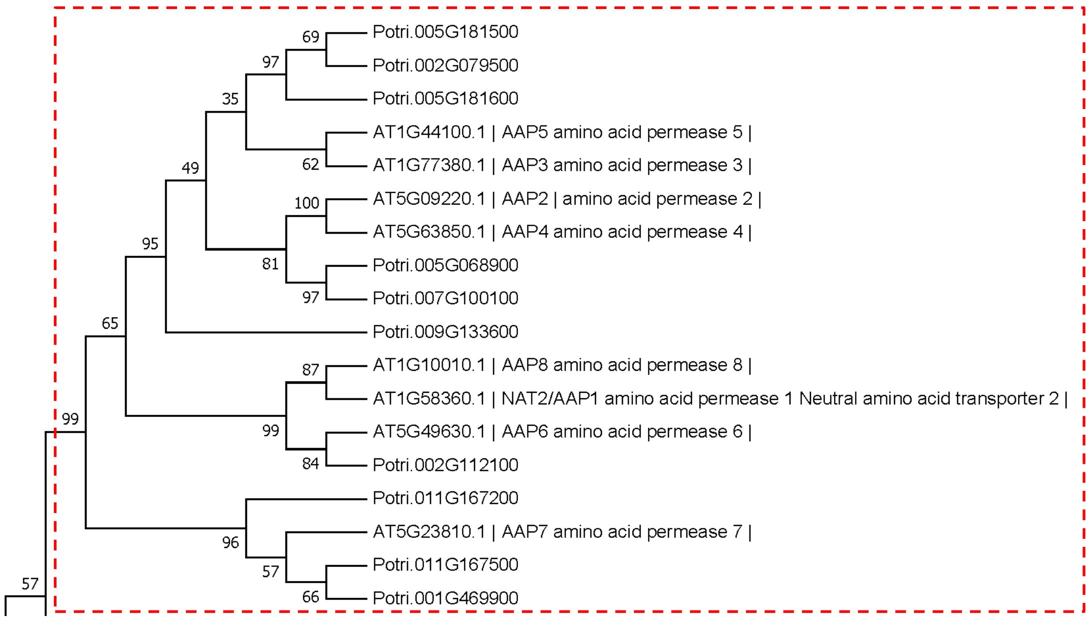

69
97
35
49
95
65
99
57
Potri.005G181500
Potri.002G079500
Potri.005G181600
AT1G44100.1 | AAP5 amino acid permease 5 |
62
AT1G77380.1 | AAP3 amino acid permease 3 |
100
AT5G09220.1 | AAP2 | amino acid permease 2 |
AT5G63850.1 | AAP4 amino acid permease 4 |
81
Potri.005G068900
97
Potri.007G100100
Potri.009G133600
87
AT1G10010.1 | AAP8 amino acid permease 8 |
AT1G58360.1 | NAT2/AAP1 amino acid permease 1 Neutral amino acid transporter 2 |
99
AT5G49630.1 | AAP6 amino acid permease 6 |
84
Potri.002G112100
Potri.011G167200
AT5G23810.1 | AAP7 amino acid permease 7 |
96
57
Potri.011G167500
66
Potri.001G469900

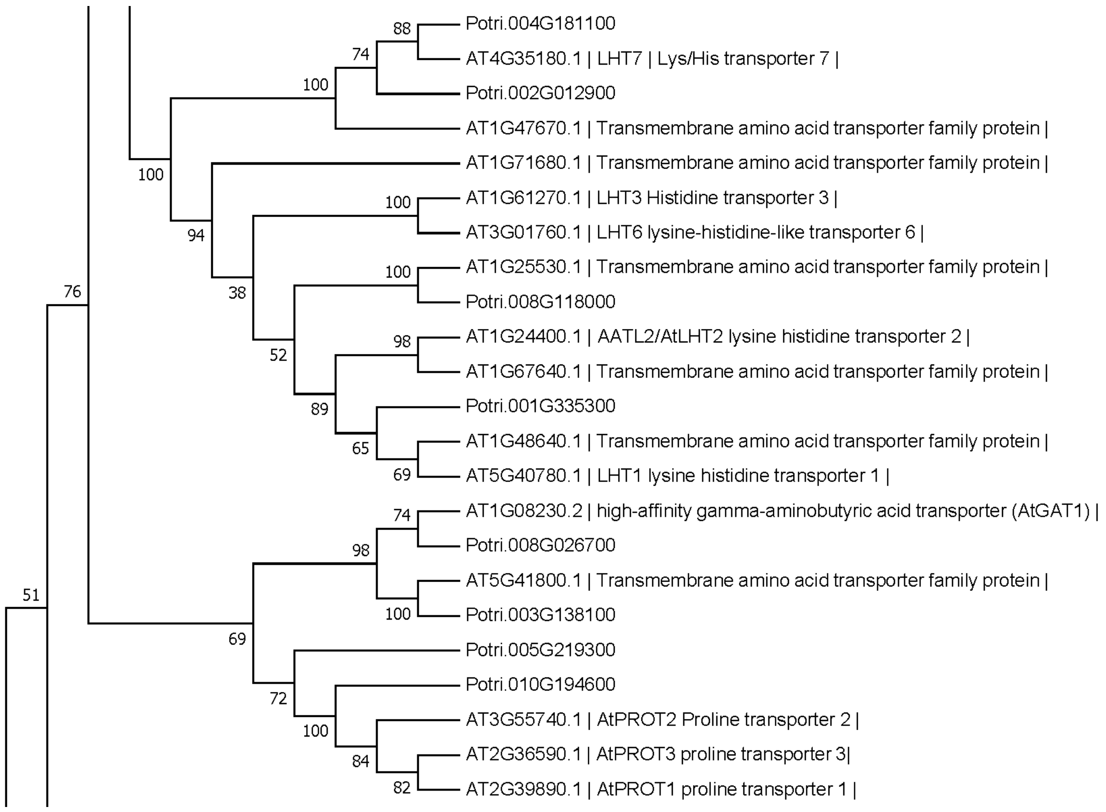
Potri.004G181100
AT4G35180.1 | LHT7 | Lys/His transporter 7 |
Potri.002G012900
AT1G47670.1 | Transmembrane amino acid transporter family protein |
AT1G71680.1 | Transmembrane amino acid transporter family protein |
AT1G61270.1 | LHT3 Histidine transporter 3 |
AT3G01760.1 | LHT6 lysine-histidine-like transporter 6 |
AT1G25530.1 | Transmembrane amino acid transporter family protein |
Potri.008G118000
AT1G24400.1 | AATL2/AtLHT2 lysine histidine transporter 2 |
AT1G67640.1 | Transmembrane amino acid transporter family protein |
Potri.001G335300
AT1G48640.1 | Transmembrane amino acid transporter family protein |
AT5G40780.1 | LHT1 lysine histidine transporter 1 |
AT1G08230.2 | high-affinity gamma-aminobutyric acid transporter (AtGAT1) |
Potri.008G026700
AT5G41800.1 | Transmembrane amino acid transporter family protein |
Potri.003G138100
Potri.005G219300
Potri.010G194600
AT3G55740.1 | AtPROT2 Proline transporter 2 |
AT2G36590.1 | AtPROT3 proline transporter 3|
AT2G39890.1 | AtPROT1 proline transporter 1 |
88
74
100
100
94
100
38
100
52
98
89
65
69
76
74
98
100
51
69
72
100
84
82

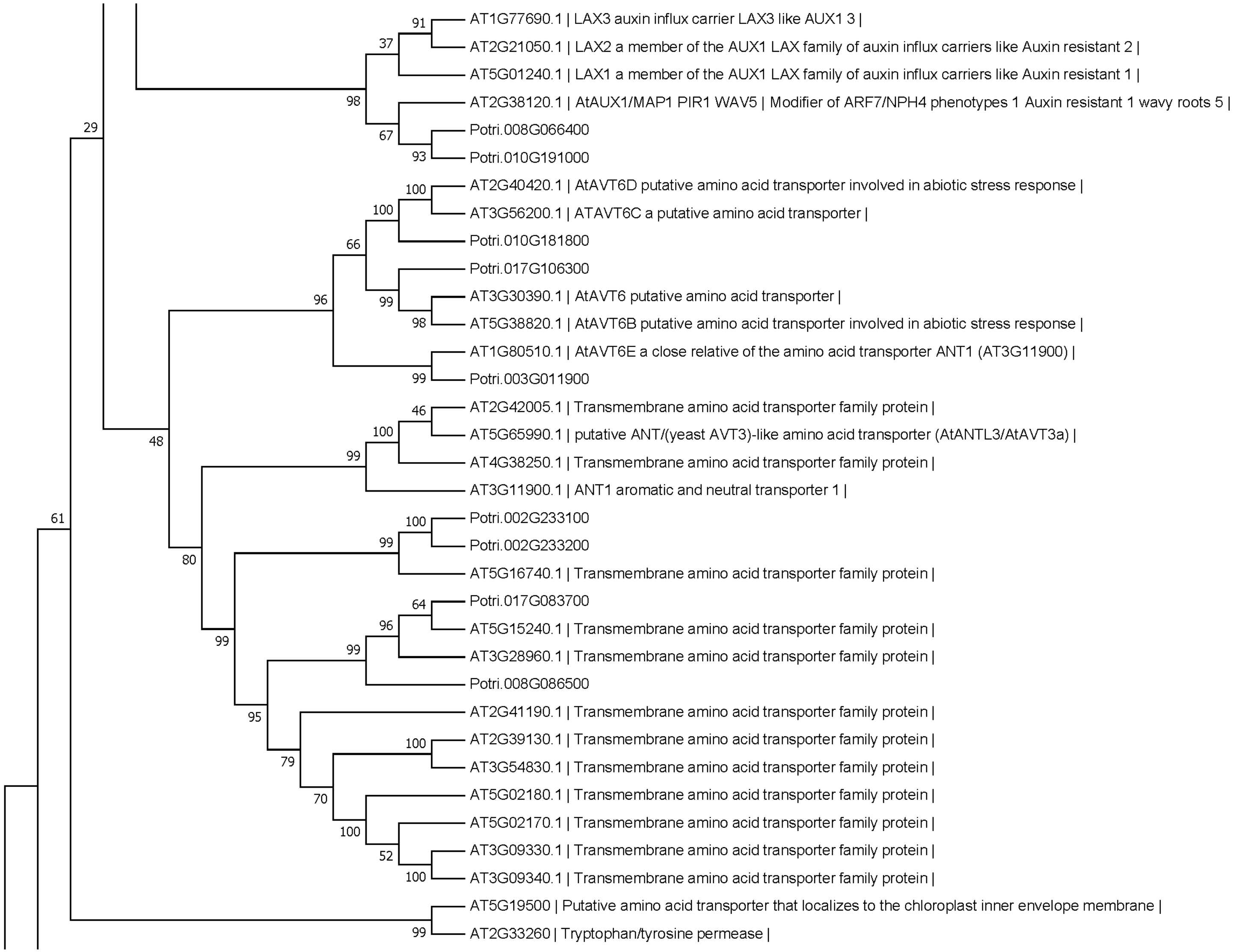
AT1G77690.1 | LAX3 auxin influx carrier LAX3 like AUX1 3 |
AT2G21050.1 | LAX2 a member of the AUX1 LAX family of auxin influx carriers like Auxin resistant 2 |
AT5G01240.1 | LAX1 a member of the AUX1 LAX family of auxin influx carriers like Auxin resistant 1 |
AT2G38120.1 | AtAUX1/MAP1 PIR1 WAV5 | Modifier of ARF7/NPH4 phenotypes 1 Auxin resistant 1 wavy roots 5 |
Potri.008G066400
Potri.010G191000
AT2G40420.1 | AtAVT6D putative amino acid transporter involved in abiotic stress response |
AT3G56200.1 | ATAVT6C a putative amino acid transporter |
Potri.010G181800
Potri.017G106300
AT3G30390.1 | AtAVT6 putative amino acid transporter |
AT5G38820.1 | AtAVT6B putative amino acid transporter involved in abiotic stress response |
AT1G80510.1 | AtAVT6E a close relative of the amino acid transporter ANT1 (AT3G11900) |
Potri.003G011900
AT2G42005.1 | Transmembrane amino acid transporter family protein |
AT5G65990.1 | putative ANT/(yeast AVT3)-like amino acid transporter (AtANTL3/AtAVT3a) |
AT4G38250.1 | Transmembrane amino acid transporter family protein |
AT3G11900.1 | ANT1 aromatic and neutral transporter 1 |
Potri.002G233100
Potri.002G233200
AT5G16740.1 | Transmembrane amino acid transporter family protein |
Potri.017G083700
AT5G15240.1 | Transmembrane amino acid transporter family protein |
AT3G28960.1 | Transmembrane amino acid transporter family protein |
Potri.008G086500
AT2G41190.1 | Transmembrane amino acid transporter family protein |
AT2G39130.1 | Transmembrane amino acid transporter family protein |
AT3G54830.1 | Transmembrane amino acid transporter family protein |
AT5G02180.1 | Transmembrane amino acid transporter family protein |
AT5G02170.1 | Transmembrane amino acid transporter family protein |
AT3G09330.1 | Transmembrane amino acid transporter family protein |
AT3G09340.1 | Transmembrane amino acid transporter family protein |
AT5G19500 | Putative amino acid transporter that localizes to the chloroplast inner envelope membrane |
AT2G33260 | Tryptophan/tyrosine permease |
91
37
98
67
93
29
100
100
66
96
99
98
99
46
100
99
48
61
100
99
80
64
96
99
99
95
100
79
70
100
52
100
99

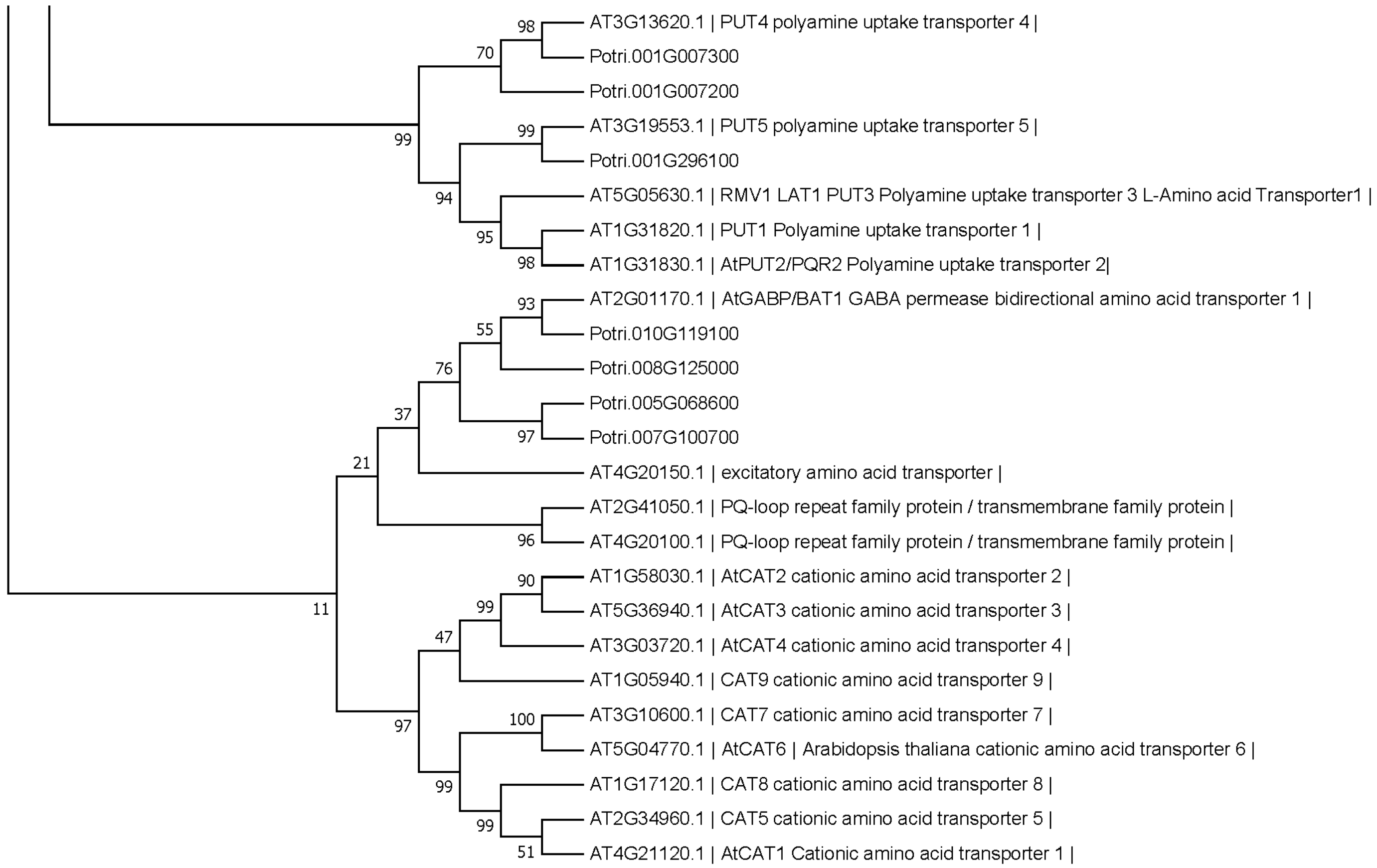

Supplemental Figure S2. The intact phylogenetic tree of 34 PtAATs and 66 AtAATs. The gene IDs of these proteins were listed in Supplemental Table 4. The subclade containing all 8 AtAATs encoding amino acid permeases (AAPs) and 10 PtAATs was enclosed in the read dashed rectangles, which was analyzed further in main text.
